# Psychometric Properties of the Greek Version of the Perinatal Anxiety Screening Scale

**DOI:** 10.7759/cureus.86979

**Published:** 2025-06-29

**Authors:** Maria Dagla, Irina Mrvoljak-Theodoropoulou, Vassilis Daglas, Evangelia Antoniou, Ioanna Mouchtari, Calliope Dagla, Dimitra Sotiropoulou, Eleni Tsolaridou, Despoina Karagianni

**Affiliations:** 1 Department of Midwifery, University of West Attica, Athens, GRC; 2 Day Center for the Care of the Mental Health of Women (Perinatal Mental Health Disorders), Non-Profit Organization “FAINARETI”, Athens, GRC; 3 Department of Psychology, National Kapodistrian University, Athens, GRC

**Keywords:** antenatal, pass, perinatal anxiety, perinatal period, postpartum, pregnancy, reliability, screening, validity

## Abstract

Background

It has been widely acknowledged in the literature that the most reliable and valid method of assessing perinatal anxiety is through reliable and valid measures, as anxiety is highly prevalent among women during the perinatal period. This study aimed to examine the psychometric properties and the validity and reliability of the Perinatal Anxiety Screening Scale (PASS) in Greek women in the perinatal period. To date, no study has been conducted in Greece assessing the psychometric properties of the PASS.

Methods

This is a retrospective study of 518 women who gave birth in Athens, Greece, between January 2016 and January 2019. The participants were followed from pregnancy up to the first postnatal year within a healthcare intervention, carried out at the Day Center for the Care of the Mental Health of Women (Perinatal Mental Health Disorders), run by the Non-Profit Organization FAINARETI. Measures used in the study were the PASS and the Edinburgh Postnatal Depression Scale (EPDS).

Results

To assess the internal reliability of the PASS, two different internal consistency indices were calculated, showing a high reliability level. The data had a positive skewing effect, indicating low reported levels of perinatal anxiety among the participants. Correlation analysis between the PASS and the EPDS showed good convergence scale validity, while conducted factor analyses demonstrated fitting construct validity.

Conclusions

These findings suggest that the four-factor PASS appears to be adequate for the Greek population of women in the perinatal period.

## Introduction

Perinatal anxiety requires early detection and treatment due to the potential long-term effects on both infants and mothers. In pregnancy and postpartum, anxiety disorders often recur or worsen for many women. Postnatal (perinatal) anxiety disorders are also more prevalent than in the general population [1]. When an anxiety disorder occurs during the perinatal period, serious consequences could follow. In addition to affecting a woman's mental health, postnatal depression also increases, as well as the risk of other disorders, together with possible adverse effects on the unborn child [2]. A study found that women with anxiety disorders are three times more likely to develop postnatal depression during pregnancy [3]. Throughout the postnatal period, mothers suffering from generalized anxiety disorders were observed to be less responsive and less engaging with their infants, were more withdrawn, and showed a less positive emotional tone toward infants, in comparison to non-anxious mothers [4].

There are estimates that between 13-25% of pregnant women and 11-20% of postpartum women experience clinically significant anxiety [5-6]. During the first trimester, when diagnostic interviews were employed, anxiety disorders accounted for 18% of all pregnancies, with a decrease to about 15% during the final two trimesters, followed by a steady decrease postnatally throughout the first year [7]. Using a meta-analysis of articles from many sources, Goodman, Watson, and Stubbs estimated a prevalence of 8.5% of anxiety disorders during pregnancy, compared with 10-15% of depression [8-9]. A systematic review found 58.5% of pregnant women suffer from antenatal anxiety symptoms, and 73.5% experience antenatal depression symptoms, respectively [10]. Several more studies confirm that nearly one-third of pregnant women may experience anxiety symptoms during pregnancy. Furthermore, antenatal anxiety appears to be the precursor to postnatal anxiety in about two-thirds of women [11-12].

Most women who experience clinically significant anxiety symptoms during pregnancy, even when they do not meet the diagnostic criteria for a specific anxiety disorder, distinguish it from anxiety experienced at other times of their lives [13-14]. Pregnant women frequently worry about their health and the health of their unborn child, as well as about labor and delivery, their finances, and their role as mothers. Pregnancy-specific anxiety is characterized by intense, persistent, and functional symptoms that differ from common worries and concerns [15].

Due to a lack of developed screening measures specifically for anxiety in perinatal women, its identification has been limited. The Perinatal Anxiety Screening Scale (PASS) was the first questionnaire that screened for a variety of anxiety disorders, as well as some typical fears unique to the perinatal stage. It was developed and evaluated based on antenatal and postnatal anxiety interviews with women attending an Australian tertiary obstetric hospital. Various anxiety disorders were assessed through designed items based on the Diagnostic and Statistical Manual of Mental Disorders (DSM) of the American Psychiatric Association (APA) and the International Classification of Diseases, Tenth Revision (ICD-10) symptoms. Results indicated that the PASS measures four domains psychometrically successfully: 1) General Worry and Specific Fears, 2) Perfectionism, Control, and Trauma, 3) Social Anxiety, and 4) Acute Anxiety and Adjustment. Validity and reliability studies of the original scale reported good internal consistency (coefficients alpha of .90, .89, .86, and .87, respectively) and a test-retest correlation coefficient of 0.74. The correlation between the PASS and other measures of depression and anxiety ranged between 0.77 and 0.83 [16].

It is crucial to have a thorough understanding of pregnancy-related anxiety and to implement preventive measures or therapeutic approaches. For the patient's treatment to be effective, it's very important to understand how severe their anxiety is [17]. Women may be given maternity and childcare services, counselling services, psycho-social therapy, or, in extreme cases, psychiatric hospitalization, depending on the severity of their symptoms. Diagnoses made by psychiatrists are the gold standard for identifying anxiety [17-18]. However, as all pregnant women can't be regularly evaluated by a psychiatrist, they may be screened for anxiety using standard questionnaires. The progression of the patient through the stages of anxiety can be tracked with the aid of a validated tool that evaluates the severity of perinatal and postnatal anxiety [19], which also makes it easier to modify the treatment strategy as necessary.

So far, several questionnaires have been created and approved for this use [20]. However, Somerville and colleagues found the PASS useful for determining the intensity of perinatal anxiety. Consequently, the PASS is a significant addition to the body of research on perinatal anxiety and has the potential to offer practitioners a significant, more accurate, and beneficial alternative option when conducting routine perinatal mental health screenings. It also offers a different approach for use in perinatal anxiety research in the future, where there has been a tendency to focus on depression, while assuming the presence of stress and anxiety, due to the lack of more specific perinatal anxiety measures [16].

Greek women have not yet been subjected to validation testing for the Perinatal Anxiety Screening Scale (PASS). Therefore, the primary goal of the current study was to validate the psychometric properties of the PASS in a sample of Greek women who underwent mental health screening from the second trimester of pregnancy to 12 months postpartum, due to the significance of assessing anxiety symptoms, particularly during the perinatal period.

## Materials and methods

Sample

The 518 women who gave birth in Athens, Greece, between January 2016 and January 2019, over four years, are the source of the data used in this study. Their average age was M = 35.90 ± 4.12, ranging from 26 to 47 years. The study participants were followed up retrospectively from pregnancy to the first year after giving birth.All of the women had taken part in a cutting-edge psychosocial health intervention that was carried out at the Day Center for the Care of the Mental Health of Women (Perinatal Mental Health Disorders), the only Day Center in the nation, run by the Non-Profit Organization FAINARETI. The Mental Health Department of the Greek Ministry of Health oversees and provides funding for this Day Center, a major community facility. All of the study's participants gave their informed oral and written consent to have their data analyzed for research purposes, and they were also made aware of their right to withdraw consent at any time. The first screening using the tool was implemented upon admission at the Day Center, approximately between the 18th and 22nd gestation week. The second one was completed during the last weeks of pregnancy, followed by perinatal anxiety screening at the 6th week postpartum. At the end of the 1st year postpartum, the same information was received via e-mail or by telephone. The Non-Profit Organization FAINARETI's Research Ethics Committee gave their approval for this study (Ref. Number 77/03.07.19).

Measures

For the Perinatal Anxiety Screening Scale (PASS) used in the present research, back translation procedures were completed. The employed scale was translated from English to Greek by translators with no prior knowledge of the research project and was then translated back to English by two additional independent translators.

Perinatal Anxiety Screening Scale - PASS

Perinatal Anxiety Screening Scale [16] is a 31-item valid, reliable, and practical screening instrument for determining the likelihood of significant anxiety in pregnant and postpartum women. Each item is scored on a 4-point Likert scale from 0 ‘’not at all’’ to 3 ‘’almost always’’, and the total score is the sum of all the individual scores, with higher scores denoting greater anxiety. Scores can be from 0 to 93, with a clinical anxiety cutoff of 26. By measuring four domains that address particular anxiety symptoms, as they manifest in perinatal women, it distinguishes between groups at high and low risk for presenting with an anxiety disorder. These domains form subscales which include: 1) General Worry and Specific Fears (10 items), 2) Perfectionism, Control, and Trauma (eight items), 3) Social Anxiety (five items), and 4) Acute Anxiety and Adjustment (eight items). With its global scores significantly correlated with the Edinburgh Postnatal Depression Scale (EPDS)'s anxiety subscale and overall EPDS scores, the PASS demonstrated adequate convergent validity. The scale's overall reliability is rated as excellent (Cronbach's alpha = 0.96). In the present study, Cronbach’s alpha reached .94. A four-factor structure that accounts for 59.37% of the total variance is the best fit.

Edinburgh Postnatal Depression Scale - EPDS

The Edinburgh Postnatal Depression Scale (EPDS) [21] is a widely used screening tool for antepartum and postpartum depression. It is a 10-item tool, graded on a 4-point Likert scale, with 0 being the lowest grade and 3 being the highest. By adding up the scores of all the items, a final score ranging from 0 to 30 points was determined. It has been suggested that a total score of 12 or higher serves as a potential depression indicator. The scale has been translated and validated for the Greek population by two different research groups [22-23], and it has demonstrated high overall internal consistency. The Cronbach's alpha for the total scale was equal to .90 in the validation study of Leonardou et al. [22], and .80 in the validation study of Vivilaki et al. [23]. The alpha coefficient in the current study was .88.

## Results

Descriptive statistics and reliability indices

Descriptive statistics and reliability indices of the scale are presented in Table 1. An internal consistency metric is used to assess the PASS's internal reliability (i.e., Cronbach's alpha and Guttman's split-half). The correlations between factors were strong, ranging between .510 and .737, while inter-item correlations were all statistically significant, ranging from .170 to .694, showing good item-to-scale homogeneity.

**Table 1:** Descriptive Statistics and Reliability Coefficients for the PASS PASS: Perinatal Anxiety Screening Scale

(N = 518)	M	SD	Min	Max	Alpha	Split-half	N
General Worry and Specific Fears	.70	.46	.23	1.25	.87	.83	10
Perfectionism, Control and Trauma	.80	.52	.29	1.36	.83	.82	8
Social Anxiety	.34	.40	.17	.52	.78	.76	5
Acute Anxiety and Adjustment	.39	.41	.27	.53	.85	.80	8
Total PASS Score	.59	.39	.17	1.36	.94	.89	31

Both forms of internal consistency coefficients were required to have values greater than .70 [24]. As shown in Table 1, Cronbach’s α coefficients for the total PASS, along with the four latent factors, were very high (from .78 to .94). Additionally, Guttman's split-half coefficients were similar, displaying a high degree of internal consistency (from .76 to .89), relating the present sample. Positively skewed data showed low perinatal anxiety levels among participants.

Factors validity

To determine whether the PASS would show the same latent factors as the theoretically expected ones, a quick screening was conducted using an exploratory factor analysis (EFA). Specifically, it was examined whether all items were sufficiently reliable and whether all four factors were present. To determine the number of factors, the Kaiser-Guttman criterion of value greater than one was used to extract the factors. The analysis was performed by the method of principal component analysis (PCA), with varimax axis rotation and Kaiser normalization. The widely used PCA method combines manifest (observed) variables into weighted linear combinations that become components, where the component correlations and component scores match exactly. Optimized weighted linear variable combinations are what it aims to produce. Because it maximizes the variance within a factor, greater loadings are increased and smaller loadings are minimized [25]. Varimax rotation is also the method that is most frequently used. An exploratory factor analysis was first carried out with the principal axis factoring method to provide starting values and to control the presence of some weak items (with unsatisfactory loadings). No items were omitted. Factor loadings are shown in Table 2.

**Table 2:** Exploratory Factor Analysis for the 31 PASS Items PASS: Perinatal Anxiety Screening Scale

(N = 518)	Factors					
	1	2	3	4	5	6
1. Worry about the baby/pregnancy	.264	.280	-.827	.226	.074	.138
2. Fear that harm will come to the baby	.244	.175	-.804	.275	-.037	.300
3. A sense of dread that something bad is going to happen	.407	.170	-.671	.334	-.105	.512
4. Worry about many things	.569	.403	-.716	.321	-.023	.298
5. Worry about the future	.497	.363	-.762	.331	.048	.220
6. Feeling overwhelmed	.713	.385	-.490	.326	.024	.409
7. Really strong fears about things, e.g., needles, blood, birth, pain, etch	.422	.262	-.291	.498	-.262	.230
8. Sudden rushes of extreme fear or discomfort	.693	.274	-.498	.469	-.109	.608
9. Repetitive thoughts that are difficult to stop or control	.634	.367	-.447	.394	.121	.721
10. Difficulty sleeping even when I have the chance to sleep	.424	.311	-.177	.231	.485	.469
11. Having to do things in a certain way or order	.317	.778	-.144	.292	.165	.225
12. Wanting things to be perfect	.195	.815	-.207	.167	.049	.042
3. Needing to be in control of things	.319	.830	-.303	.195	.015	.163
14. Difficulty stopping checking or doing things over and over	.362	.743	-.318	.405	.037	.339
15. Feeling jumpy or easily startled	.554	.481	-.426	.455	-.144	.384
16. Concerns about repeated thoughts	.488	.396	-.453	.461	-.016	.713
17. Being 'on guard' or needing to watch out for things	.385	.494	-.499	.297	-.083	.388
18. Upset about repeated memories, dreams or nightmares	.295	.302	-.318	.352	.118	.732
19. Worry that I will embarrass myself in front of others	.374	.340	-.338	.807	.169	.184
20. Fear that others will judge me negatively	.478	.381	-.307	.762	.126	.199
21. Feeling uneasy in crowds	.318	.193	-.219	.787	.099	.285
22. Avoiding social activities because I might be nervous	.378	.219	-.266	.712	.195	.416
23. Avoiding things which concern me	.324	.176	-.234	.388	.671	.184
24. Feeling detached like you're watching yourself in a movie	.411	.273	-.221	.468	.403	.473
25. Losing track of time and can't remember what happened	.481	.284	-.157	.418	.340	.060
26. Difficulty adjusting to recent changes	.691	.280	-.307	.338	.154	.136
27. Anxiety getting in the way of being able to do things	.745	.345	-.342	.377	.243	.394
28. Racing thoughts make it hard to concentrate	.690	.319	-.365	.455	.243	.607
29. Fear of losing control	.682	.392	-.338	.521	.055	.311
30. Feeling panicky	.632	.291	-.353	.409	-.108	.600
31. Feeling agitated	.755	.331	-.361	.443	.090	.389

According to the Kaiser Meyer Olkin criterion (KMO = .94) for the PASS, the sample was suitable for further analysis, as well as the table of interrelationships of the 31 items, according to the Bartlett criterion (χ² = 7555.57, df = 465, p<.001). Despite the theoretically expected four factors, six factors emerged, to which 59.10% of the variance of the PASS is attributed. Therefore, relating to the current study sample, factor loadings did not function as anticipated in the theory. The first factor explains 35.86% of the total variance and, relating to the study sample, consists of 10 items (seven items accounted for the factor ‘’Acute Anxiety and Adjustment’’, two items for the factor ‘’General Worry and Specific Fears’’, and one item for ‘’Perfectionism, Control and Trauma’’). The second factor consists of four items, accounted for the factor ‘’Perfectionism, Control and Trauma’’, and explains 6.27% of the total variance. The third factor consists of six items (five items of the factor ‘’General Worry and Specific Fears’’ and one item of the factor ‘’Perfectionism, Control and Trauma’’), and explains 5.98% of the total variance.

Next, the fourth factor explains 4.35% of the total variance and consists of five items (four items accounted for the factor ‘’Social Anxiety’’ and one item for the factor ‘’General Worry and Specific Fears’’). The fifth factor consists of two items (one item of the factor ‘’Social Anxiety’’ and one item of the factor ‘’General Worry and Specific Fears’’) and explains 3,41% of the total variance. The sixth and last factor explains 3.24% of the total variance of the PASS and consists of four items (two items of the factor ‘’Perfectionism, Control and Trauma’’, one item of ‘’General Worry and Specific Fears’’, and one item accounted for the factor ‘’Acute Anxiety and Adjustment’’). In addition, 35 cross-loadings were observed, but such items were accounted for factors with a higher value (Table 2).

Confirmatory factor analyses (CFA) were used for the prototype scale in the following phase [26]. It gives the impression that research is conducted in cycles when the same set of data is used for exploratory and confirmatory factor analysis. In contrast, correlations (loadings) of items within a factor to which they belong are maximally controlled in a confirmatory analysis, while loadings of factors to which items do not belong are treated as zero values and are fully controlled [27]. Models, on the other hand, differ in that they permit non-zero correlations between items and all data-related dimensions when performing an exploratory factor analysis or a principal component analysis. Due to these facts, both the results of the confirmatory and exploratory analyses are presented here, with the confirmatory approach receiving superior attention and significance.

The original four-factor structural model, χ²(428) = 1828.755, p<.001, χ²/df = 4.273, Root Mean Square Error of Approximation (RMSEA) = .080 (confidence interval: .076 - .083), Root Mean Square Residual (RMR) = .032, Goodness of Fit Index (GFI) = .792, Comparative Fit Index (CFI) = .807, Tucker-Lewis Index (TLI) = .790, Akaike Information Criterion (AIC) = 1964.755., was not entirely supported by our data, as can be seen in Table 3. However, after model modification, with 31 error covariance (e2-e5, e3-e4, e6-e7, e6-e8, e6-e9, e6-e10, e7-e8, e7-e9, e7-e10, e8-e9, e8-e10, e9-e10, e13-e14, e13-e15, e13-e16, e13-e17, e13-e18, e14-e15, e14-e16, e14-e17, e14-e18, e15-e16, e15-e17, e15-e18, e16-e17, e16-e18, e17-e18, e22-e23, e20-e21, e24-e31, & e27-e28), which differ from zero and strictly within factors, and which were identified through the modification indices, the model of the PASS with 31 items, χ²(402) = 919.918, p<.001, χ²/df = 2.288, RMSEA = .050 (confidence intervals .046 - .054), RMR = .019, GFI = .900, CFI = .929, TLI = .917, AIC = 1107.918, was fully confirmed by our data.

**Table 3:** Confirmatory Factor Analysis of the PASS M1: independence model, M2: four-factor model, M3: modified four-factor model, including error covariance estimates strictly within factors, PASS: Perinatal Anxiety Screening Scale, RMSEA: Root Mean Square Error of Approximation, RMR: Root Mean Square Residual, GFI: Goodness of Fit Index, CFI: Comparative Fit Index, TLI: Tucker-Lewis Index, AIC: Akaike Information Criterion.

	M1	M2	M3
χ²	7722.367	1828.755	919.918
df	465	428	402
p	p<.001/>	p<.001/>	p<.001/>
χ²/df	16.607	4.273	2.288
RMSEA [90% CI]	.174 [.170, .177]	.080 [.076, .083]	.050 [.046, .054]
RMR	.149	.032	.019
GFI	.218	.792	.900
CFI	.000	.807	.929
TLI	.000	.790	.917
AIC	7784.367	1964.755	1107.918

**Figure 1:**
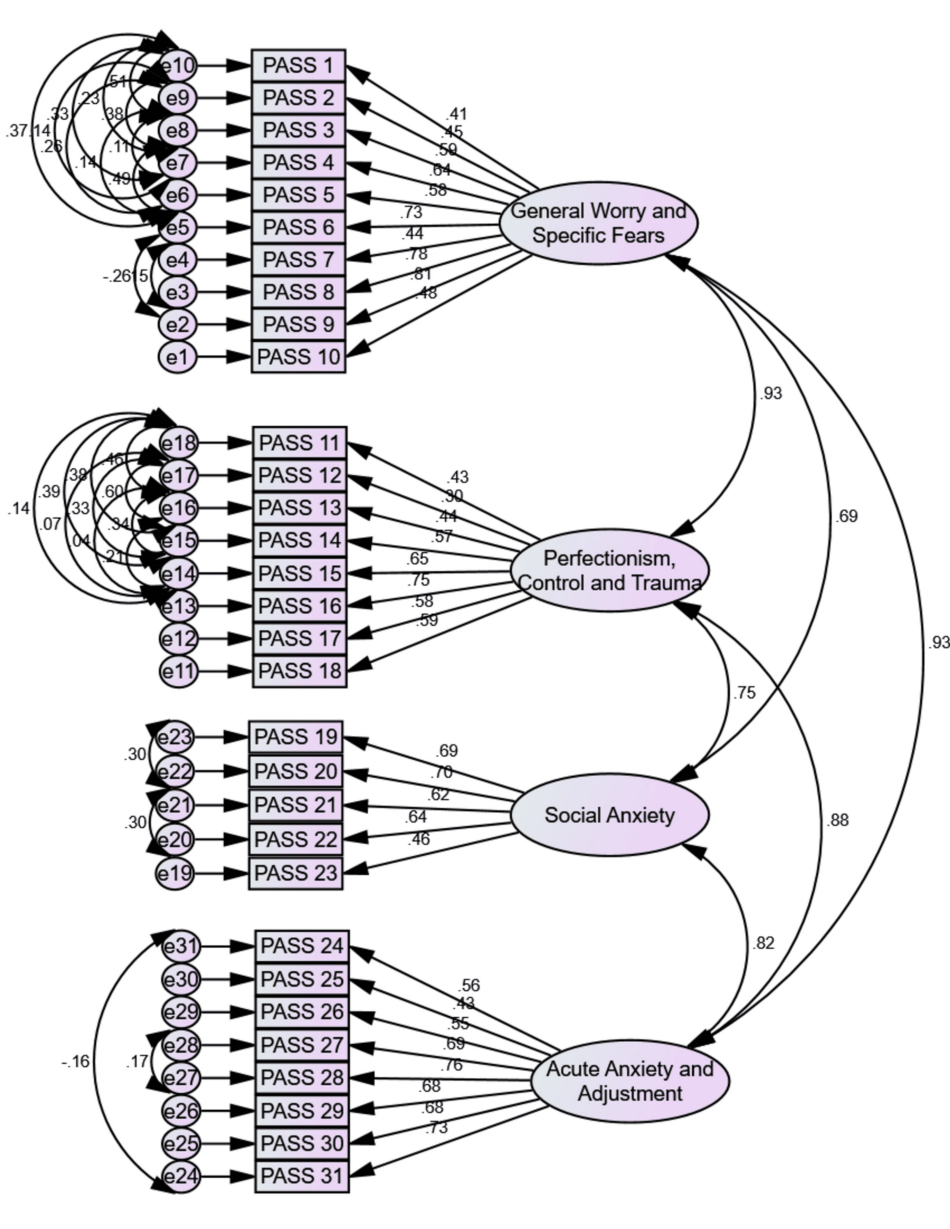
Confirmatory Factor Analysis Outcomes: Graphic Representation of the PASS Factorial Model PASS: Perinatal Anxiety Screening Scale Figure credits: Authors.

Given that confirmatory factor analysis can identify error covariances indicating that two measures covariate for reasons other than the shared factor's influence, such as method effects [28], it was decided to allow for those error covariances to differ for the best-fitting model. Furthermore, it can be observed that RMSEA, RMR, AIC, and χ²/df decreased, while GFI, TLI, and CFI increased. The solution could have been stabilized by removing some items, but doing so would have had a negative impact on the factors' theoretically expected validity, making the solution unjustifiable from a theoretical standpoint. In any case, as suggested by the scale's designers, the PASS adheres to the original dimensions presented in the scale (Figure 1), since the current study results show that the four-factor structure of the PASS has a perfect fit, and appears to be adequate for the Greek sample of women.

Scale validity

The convergent scale validity was evaluated using the Spearman rank correlation coefficient, in light of the aforementioned scale skewness. In the context of this study, the relationship between the EPDS and the PASS was examined to assess the validity of the PASS, and results showed a significant positive correlation. Specifically, the correlation was strong (ρ = .717), demonstrating that perinatal anxiety was significantly positively correlated with perinatal depression. According to the sample used in the current study, this finding suggests very good convergent scale validity.

## Discussion

The purpose of this study was to assess the psychometric characteristics of the PASS in Greek women, the first such study in Greece. To evaluate the internal consistency and homogeneity of the PASS, Cronbach's alpha, Guttman's split-half coefficient, as well as factors and item-total correlations, were calculated. Construct validity was assessed through exploratory factor analysis (EFA) to determine if the four-factor scale appears in the present study population. In addition to the EFA, confirmatory factor analyses (CFA) were conducted to estimate whether the fit of the four-factor model as found in the literature is valid. Furthermore, the criterion of convergent validity as measured by Spearman's correlation coefficient was examined by comparing the PASS total score with the EPDS.

The overall conclusion is that the results demonstrate a robust PASS factor structure concerning the sample used in the current study. The robustness of the analyses, as measured by Cronbach's Guttman’s split-half coefficients (Cronbach's alpha = .94, Guttman's split-half = .89), the inter-factor correlation (ranging between. 510 and .737), as well as inter-item correlation (ranging between 170 and .694), demonstrated that the PASS is highly consistent and reliable, with acceptable homogeneity. The validity of the scale was also statistically significant with a convergent validity of .717, suggesting a significant positive correlation between anxiety and depression in peri-natal pregnancy. Burns and Eidelson [29] suggested that any valid and reliable measure of depression and anxiety should be correlated at .70.

Furthermore, the present study provided empirical evidence for the construct validity of the PASS, with a six-factor structure, demonstrating some distinctions in EFA factor loadings, compared to the scale suggested in the theory, which is likely indicative of cultural perception differences. However, the most important finding is that, according to the CFA, the PASS’s four-factor structure is sufficient for the Greek women’s sample, since the prototype model of the PASS with 31 items was fully confirmed by our data.

With the high reliability of the PASS, measured in the research throughout several countries, there were no factorial constructs found exactly as in the prototype scale, by applying exploratory factor analyses. Specifically, slightly different factor loadings of the four identified factors were observed in Turkish [30], Bangladeshi [31], Iranian [32], Sri Lankan [33], Brazilian [34], Persian [35], Italian [36] and Arabic version of PASS [37], while the Lebanese EFA of the scale emerged with seven factors [38]. In studies, this is common, especially if many years pass between analyses, or if the populations differ in terms of mental health characteristics [39-40]. It is also likely that cultural aspects play a role in the interpretation of translations; they may be interpreted slightly differently than the original versions.

Close to our findings, obtained by confirmatory factor analyses that confirmed a perfect four-factor model fit, is the Sri-Lankan research demonstrating the factorial model close to the good fit indices [33], while in the Brazilian research, several fit indices appeared to be lower than expected for the comprehensive model confirmation [34]. Difficulties in the four-factor model confirmation were found in the Turkish study [31], as well as in the Italian study, showing inadequate fit indices for the tested factors, as in the prototype PASS [36]. Therefore, to our knowledge, the four-factor CFA model that emerged in our study is the only one that was entirely confirmed by our data, showing that the PASS could be administered in Greece as first created by the authors. 

The finding of a significant and positive correlation between the scale and depression measures [16] was not surprising, since, according to the literature, anxiety and depression commonly co-occur. It has been estimated that up to 63% of adults diagnosed with anxiety disorders also meet the diagnostic criteria for depression [41]. It has been shown that pregnant women are more likely to suffer from depression and anxiety co-morbidly than women who suffer from depression and anxiety independently [42]. A causal factor shared by depression and anxiety has been suggested by Burns and Eidelson [29] to explain the significant correlations between the two.

Being the first validation study of the scale in Greece, our study is particularly significant because the PASS was validated against the gold standard of psychosomatic diagnoses of anxiety disorders. As well as validating, proving reliable, and maintaining homogeneity, the PASS performed well when compared with the prototype four-factor model. The limitation of the study was that the sample consisted mostly of Greek antenatal women living in urban areas.

Due to the high correlation between items, it may be advantageous for future studies to create a condensed version of the PASS, which may facilitate a more user-friendly experience in a bustling community practice environment. Finally, this tool may be beneficial in future research to evaluate perinatal anxiety among the Greek female population. We recommend standardizing clinical practice by incorporating this questionnaire.

## Conclusions

There are some differences in EFA loadings in the present study, as compared to the scale suggested by the theory, which may reflect cultural perceptions differences. The most important finding, however, is based on the CFA, demonstrating that the four-factor structure of the PASS is acceptable for the Greek women's sample, since the prototype model of the PASS with 31 items was fully confirmed by our data.

## References

[1] Ross LE, McLean LM (2006). Anxiety disorders during pregnancy and the postpartum period: a systematic review. J Clin Psychiatry.

[2] Heron J, O'Connor TG, Evans J, Golding J, Glover V (2004). The course of anxiety and depression through pregnancy and the postpartum in a community sample. J Affect Disord.

[3] Sutter-Dallay AL, Murray L, Dequae-Merchadou L, Glatigny-Dallay E, Bourgeois ML, Verdoux H (2011). A prospective longitudinal study of the impact of early postnatal vs. chronic maternal depressive symptoms on child development. Eur Psychiatry.

[4] Stein A, Craske MG, Lehtonen A (2012). Maternal cognitions and mother-infant interaction in postnatal depression and generalized anxiety disorder. J Abnorm Psychol.

[5] Woods SM, Melville JL, Guo Y, Fan MY, Gavin A (2010). Psychosocial stress during pregnancy. Am J Obstet Gynecol.

[6] Kang YT, Yao Y, Dou J (2016). Prevalence and risk factors of maternal anxiety in late pregnancy in China. Int J Environ Res Public Health.

[7] Dennis CL, Falah-Hassani K, Shiri R (2017). Prevalence of antenatal and postnatal anxiety: systematic review and meta-analysis. Br J Psychiatry.

[8] Goodman JH, Watson GR, Stubbs B (2016). Anxiety disorders in postpartum women: a systematic review and meta-analysis. J Affect Disord.

[9] Tahirkheli NN, Cherry AS, Tackett AP, McCaffree MA, Gillaspy SR (2014). Postpartum depression on the neonatal intensive care unit: current perspectives. Int J Womens Health.

[10] Ferreira CR, Orsini MC, Vieira CR, do Amarante Paffaro AM, Silva RR (2015). Prevalence of anxiety symptoms and depression in the third gestational trimester. Arch Gynecol Obstet.

[11] Wenzel A, Haugen EN, Jackson LC, Robinson K (2003). Prevalence of generalized anxiety at eight weeks postpartum. Arch Womens Ment Health.

[12] O'Connor TG, Heron J, Golding J, Beveridge M, Glover V (2002). Maternal antenatal anxiety and children's behavioural/emotional problems at 4 years. Report from the Avon Longitudinal Study of Parents and Children. Br J Psychiatry.

[13] Huizink AC, Mulder EJ, Robles de Medina PG, Visser GH, Buitelaar JK (2004). Is pregnancy anxiety a distinctive syndrome?. Early Hum Dev.

[14] Phillips J, Sharpe L, Matthey S, Charles M (2009). Maternally focused worry. Arch Womens Ment Health.

[15] Thorsness KR, Watson C, LaRusso EM (2018). Perinatal anxiety: approach to diagnosis and management in the obstetric setting. Am J Obstet Gynecol.

[16] Somerville S, Dedman K, Hagan R (2014). The Perinatal Anxiety Screening Scale: development and preliminary validation. Arch Womens Ment Health.

[17] Austin M, Highet N (2011). Clinical Practice Guidelines for Depression and Related Disorders-Anxiety, Bipolar Disorder and Puerperal Psychosis in the Perinatal Period: A Guideline for Primary Care Health Professionals. https://web.archive.org/web/20121010051517/http:/www.beyondblue.org.au/index.aspx?link_id=6.1246.

[18] Fairbrother N, Young AH, Janssen P, Antony MM, Tucker E (2015). Depression and anxiety during the perinatal period. BMC Psychiatry.

[19] Ronk FR, Korman JR, Hooke GR, Page AC (2013). Assessing clinical significance of treatment outcomes using the DASS-21. Psychol Assess.

[20] Askarizadeh G, Karamoozian M, Darekordi A (2017). Validation of Iranian version of Pregnancy Related Anxiety Questionnaire. Int J Prev Med.

[21] Cox JL, Holden JM, Sagovsky R (1987). Detection of postnatal depression. Development of the 10-item Edinburgh Postnatal Depression Scale. Br J Psychiatry.

[22] Leonardou AA, Zervas YM, Papageorgiou CC (2009). Validation of the Edinburgh Postnatal Depression Scale and prevalence of postnatal depression at two months postpartum in a sample of Greek mothers. J Reprod Infant Psychol.

[23] Vivilaki VG, Dafermos V, Kogevinas M, Bitsios P, Lionis C (2009). The Edinburgh Postnatal Depression Scale: translation and validation for a Greek sample. BMC Public Health.

[24] Nunnally JC, Bernstein IH (1994). The assessment of reliability. Psychometric Theory.

[25] Osborne JW (2014). Best Practices in Exploratory Factor Analysis. https://www.researchgate.net/publication/265248967_Best_Practices_in_Exploratory_Factor_Analysis.

[26] Hu L, Bentler PM (1999). Cutoff criteria for fit indexes in covariance structure analysis: conventional criteria versus new alternatives. Struct Equat Model.

[27] van Prooijen JW, van der Kloot WA (2001). Confirmatory analysis of exploratively obtained factor structures. Educ Psychol Measur.

[28] Galanaki EP, Mylonas K, Vogiatzoglou PS (2015). Evaluating voluntary aloneness in childhood: initial validation of the Children’s Solitude Scale. Eur J Dev Psychol.

[29] Burns DD, Eidelson RJ (1998). Why are depression and anxiety correlated? A test of the tripartite model. J Consult Clin Psychol.

[30] Yazici E, Pek TM, Yuvaci HU (2018). Perinatal Anxiety Screening Scale validity and reliability study in Turkish (PASS-TR validity and reliability). Psychiatry Clin Psychopharmacol.

[31] Yasmin F, Islam S (2018). Adaptation of the Perinatal Anxiety Screening Scale in the Bangladeshi context. Psychol Res Int J.

[32] Barzgar-Molan S, Farshbaf-Khalili A, Asghari JM, Babapour J, Yavarikia P (2020). Psychometric properties of the Iranian version of a perinatal anxiety screening scale in Iranian perinatal population: a methodological study. Cresc J Med Biol Sci.

[33] Priyadarshanie MN, Waas MD, Goonewardena CS, Balasuriya A, Senaratna BC, Fernando DM (2020). Sinhala translation of the Perinatal Anxiety Screening Scale: a valid and reliable tool to detect anxiety disorders among antenatal women. BMC Psychiatry.

[34] Barros M, Aguiar M, Macedo A, Pereira AT (2021). Validity and reliability of the perinatal anxiety screening scale in a brazilian sample of pregnant women. Eur Psychiatry.

[35] Amiri P, Bahaadinbeigy K, Asadi F, Rahmati S, Mazhari S (2022). Validation of the Persian version of the Perinatal Anxiety Screening Scale (PASS) among antenatal and postnatal women. BMC Pregnancy Childbirth.

[36] Koukopoulos A, Mazza C, De Chiara L (2021). Psychometric properties of the Perinatal Anxiety Screening Scale Administered to Italian women in the perinatal period. Front Psychiatry.

[37] Jradi H, Alfarhan T, Alsuraimi A (2020). Validation of the Arabic version of the Perinatal Anxiety Screening Scale (PASS) among antenatal and postnatal women. BMC Pregnancy Childbirth.

[38] Hobeika E, Malaeb D, Hobeika E (2020). Postpartum depression and anxiety among Lebanese women: correlates and scales validation. BMC Psychiatry.

[39] Comparelli A, Savoja V, Kotzalidis GD (2011). Factor-structure of the Italian version of the Scale Of Prodromal Symptoms (SOPS): a comparison with the English version. Epidemiol Psychiatr Sci.

[40] Kotzalidis GD, Solfanelli A, Piacentino D (2017). The Italian version of the 92-item Prodromal Questionnaire: Concurrent validity with the SIPS and factor analysis in a sample of 258 outpatients aged 11-36years. Schizophr Res.

[41] Lamers F, van Oppen P, Comijs HC (2011). Comorbidity patterns of anxiety and depressive disorders in a large cohort study: the Netherlands Study of Depression and Anxiety (NESDA). J Clin Psychiatry.

[42] Field T, Diego M, Hernandez-Reif M (2010). Comorbid depression and anxiety effects on pregnancy and neonatal outcome. Infant Behav Dev.

